# Precapture of CO_2_ and Hydrogenation into Methanol on Heterogenized Ruthenium and Amine‐Rich Catalytic Systems

**DOI:** 10.1002/open.202300060

**Published:** 2023-05-31

**Authors:** Anthony E. Szego, Tamara L. Church, Zoltán Bacsik, Aleksander Jaworski, Latif Ullah, Niklas Hedin

**Affiliations:** ^1^ Department of Materials and Environmental Chemistry Stockholm University 10691 Stockholm Sweden; ^2^ Current address: Cambrex Karlskoga AB 691 33 Karlskoga Sweden

**Keywords:** carbon dioxide, heterogeneous catalysis, hydrogenation, methanol, ruthenium

## Abstract

A heterogenized alternative to the homogeneous precapture of CO_2_ with amines and subsequent hydrogenation to MeOH was developed using aminated silica and a Ru‐MACHO^TM^ catalyst. Commercial mesoporous silica was modified with three different amino‐silane monomers and used as support for the Ru catalyst. These composites were studied by TEM and solid‐state NMR spectroscopy before and after the catalytic reaction. These catalytic reactions were conducted at 155 °C at a H_2_ and CO_2_ pressures of 75 and 2 bar, respectively, with the heterogeneous system (gas‐solid) being probed with gas‐phase infrared spectroscopy used to quantify the resulting products. High turnover number (TON) values were observed for the samples aminated with secondary amines.

## Graphical abstract

Heterogeneous catalysts were prepared and tested in the hydrogenation of CO_2_ to methanol. These are based on the concept of precapture and transformation used in homogeneous systems.

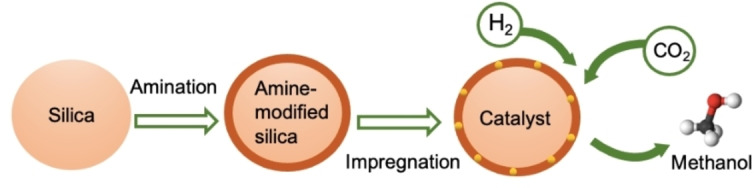

## Introduction

Global warming is a problem that has been and is tackled by numerous policy approaches, technological inventions and behavioral changes. Most energy‐system evaluations assume it is necessary for the massive amounts of industrially emitted CO_2_ to be eliminated by means of carbon capture and storage.^[1]^ However, alternative processes, in which the captured CO_2_ is recycled back into fuels and materials, are actively researched.[^2^, ^3^] Although converting captured CO_2_ to useful molecules is appealing, the high stability of CO_2_ means that its transformation requires some combination of high energy input, high temperatures and pressures, and the use of highly reactive compounds such as H_2_.

The production, storage and distribution of renewable H_2_ is itself a challenging task; hence the extent to which it can be used to convert CO_2_ will depend on economic factors such as market size, availability of renewable feedstock and environmental impact of the H_2_‐generation technologies chosen.^[4]^ H_2_ can be generated from the electrolysis of H_2_O using renewable energy sources such as solar, wind, etc.^[5]^ Many fuel‐related C_1_ products such as CH_4_, methanol (CH_3_OH), and HCOOH can be obtained by treating CO_2_ with H_2_.[^6^, ^7^, ^8^] Among CO_2_ hydrogenation products, CH_3_OH is one of the most attractive because it can be used as a drop‐in liquid fuel for already‐established internal combustion engines and direct methanol fuel cells (DMFC).[^9^, ^10^] Moreover, CH_3_OH is a viable H_2_ storage medium (12.5 wt % H_2_) and a convenient chemical feedstock for a broad array of chemicals and products including ethylene and propylene (through the methanol‐to‐olefin process).^[11]^ CH_3_OH is already one of the most important building blocks and solvents in the chemical industry, with an annual production of 98 million tons in 2019.^[12]^

Industrially CH_3_OH is currently synthesized from syngas (CO, H_2_, and CO_2_) over Cu/ZnO/Al_2_O_3_‐type heterogeneous catalysts under high pressures (>60 atm) and elevated temperatures (>200 °C);[^13^, ^14^, ^15^] however, CO_2_ has also been converted to CH_3_OH using homogeneous catalysts.^[16]^ The most effective discrete catalysts include ruthenium‐based pincer complexes,[^17^, ^18^, ^19^, ^20^] but other complexes are also promising.^[21]^ A precapture of the CO_2_ in the form of carbamates, bicarbonates, and maybe carbamic acids with amines or other bases and two‐phase systems enhances the overall conversion.[^22^, ^23^, ^24^, ^25^] This can be achieved both in homogeneous and heterogeneous systems. In contrast to homogeneous catalysts, heterogeneous catalysts play an important role in industrial chemical production.^[26]^ They are preferred because of their robustness and lower operational cost, in particular through their easier recovery/separation from the products allowing chemical processes to be streamlined.

**Figure 1:**
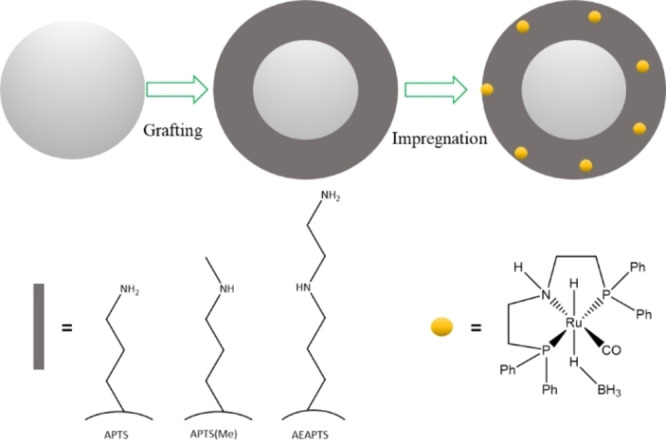
Graphic description of the heterogenized system consisting of aminated silica and the Ru‐MACHO^TM^ catalyst used for the hydrogenation of CO_2_ at 155 °C and H_2_ and CO_2_ pressures of 75 and 2 bar respectively.

The precapture approach in homogenous Ru‐based systems[^17^, ^18^, ^19^, ^20^] prompted us to hypothesize, prepare and test a heterogeneous Ru‐based catalytic system that also contained tethered amines, which jointly enabled the hydrogenation of CO_2_ to CH_3_OH. Our catalyst systems were built by grafting amines onto commercial mesoporous silica, then loading a homogeneous ruthenium catalyst (Ru‐MACHO^TM^), as illustrated in Figure 1. Solid adsorbents used as part of CO_2_ hydrogenation have previously been reported by Prakash et al.^[27]^

## Results and Discussion

The composites were prepared by first grafting one of three aminosilanes, namely (3‐aminopropyl)triethoxysilane (APTS), (3‐(methylamino)propyl)trimethoxysilane (APTMS(Me)) or (3‐(2‐aminoethylamino)propyltriethoxysilane (AEAPTS), on chromatographic silica. Then, the Ru‐MACHO^TM^ catalyst was deposited on the modified silica to produce a composite that was tested in a custom reactor for the conversion of CO_2_ into CH_3_OH under high H_2_ pressure (75 bar of H_2_ and 2 bar of CO_2_) at a relatively low temperature of 155 °C. CO_2_ adsorption isotherms of the catalysts, measured at 0 °C (Figure S2, Supporting Information), showed that all the samples took up approximately 1 mmol of CO_2_/g of material. This is a gas‐solid system, so the volatile products were analyzed using dispersive gas‐phase IR spectroscopy, which is a non‐destructive technique that can quantify multiple components across a relatively broad concentration range with a single measurement, and can provide detection limits down to the parts‐per‐billion (ppb) range for many components. This technique is especially useful here because of the presence of CO in the gas mixture, as it can be difficult to detect and quantify using mass spectrometry, as the m/z=28 fragment could come from CO_2_ or from the carrier gas used).^[28]^

The CO_2_ conversion and the TON reached 32 % and 3800 for Ru_AEAPTS (Table 1). Here, the TON denotes the amount of MeOH formed in relation to the amount of catalyst (the equation used is shown in the Supporting Information). Thus Ru_AEAPTS converted almost three times as much CO_2_ to MeOH as Pd nanoparticles on monoaminated silica,^[29]^ although Ru_AEAPTS_2 (with half the loading of immobilized molecular catalyst) converted less CO_2_ than either of these systems. CO_2_ that has been immobilized on secondary rather than primary amines is hydrogenated to CH_3_OH with better selectivity over Ru‐based homogenous catalysts,^[25]^ consistent with our findings that aminated supports bearing secondary amines were not only more selective but also more active than Ru_APTS. Computational studies of homogenous catalytic systems have established that the C−N breakage in dimethylaminomethanol was more energetically favored than in methylaminomethanol, meaning that secondary amines capture CO_2_ less strongly, and could be preferred over primary amines in the precapture‐and‐hydrogenation cycle of CO_2_.^[25]^

**Table 1:** Results from the catalytic hydrogenation of CO_2_ to methanol using a heterogenized catalytic system with aminated silica and a supported Ru‐MACHO^TM^ molecular catalyst.^[a]^

		Products				Selectivity to MeOH [%]	TON (mol(MeOH)/mol(Ru_MACHO^TM^))
	Amine type	MeOH	CO	Ammonia	Conversion		
Catalyst		[μmol]	[μmol]	[μmol]	[%]^[b]^		
Ru_AEAPTS	Primary and secondary	58	72	5.8	32	45	3800
Ru_AEAPTS_2 (lower loading)	Primary and secondary	16	1.2	6.5	9.0	93	3000
Ru_APTS	Primary	0.83	3.7	0.58	0.45	19	98
Ru_APTS(Me)	Secondary	60	22	1.1	33	73	3800

[a] The reactor was first purged using a Schlenk line, then sealed and loaded with the appropriate amount of catalyst inside a glovebox. Once loaded, the reactor was submitted to 2 bar CO_2_ for 2 h at 25 °C. The pressure was then switched to 75 bar H_2_ and the temperature was increased to 155 °C. The reactor was held at that temperature for 40 h, then cooled to room temperatures and the gases were extracted with vacuum and N_2_ flushing into a gas‐phase IR cell and analyzed (see Supporting Information for details). [b] The conversion is calculated in relation to the 2 bar of initial CO_2_.

The conversion of CO_2_ into CH_3_OH on aminated silica with a heterogenized molecular catalyst is a complex multistage process that requires both catalyst components; neither silica modified with APTS(Me) but without Ru‐MACHO^TM^, nor Ru‐MACHO^TM^ supported on unmodified silica catalyzed the hydrogenation of CO_2_. Precapture of CO_2_ on aminated supports appears to play an important part in its conversion into CH_3_OH and secondary amines were advantageous in terms of overall activity.^[30]^

In addition to the desired MeOH, two byproducts were observed from the hydrogenation: CO, which can be formed through the reverse water‐gas shift reaction^[31]^ and ammonia, which may have formed from the decomposition of the amine groups on the aminated support. We observed a competition between the hydrogenation of CO_2_ and the reverse water‐gas shift reaction, as is usual in these types of systems,^[32]^ and the product distribution depends on both the amine that was grafted on the support and the loading of Ru. This variation suggests that the interface between the adsorbed species and the active center is important in the control of the selectivity of the catalysts, in agreement with the recently reported findings of Pazdera et al. regarding the use of Pd nanoparticles on silica aminated with primary amines for the hydrogenation of CO_2_ to CH_3_OH.^[29]^ They concluded that the activity of their catalyst was given by the interface between Pd nanoparticles and the aminated support, and that a higher concentration of active centers led to a distribution of products leaning towards the formation of CH_3_OH over CO. In our study, it was clear that secondary amines were much more advantageous than primary amines to the hydrogenation of precaptured CO_2_ to CH_3_OH. Both the catalytic systems in this study and those of Pazdera et al.^[29]^ focusing on Pd nanoparticles and monoaminated catalytic systems can catalyze the synthesis of CH_3_OH at temperatures that are significantly lower than for the commercially used catalysts.[^13^, ^14^, ^15^, ^33^] Clearly, the amine‐modified silica support plays a key role in the selectivity and activity of the catalyst, and secondary amines are preferred for the precapture of CO_2_. In our case, the aminated supports with secondary amines, that is, Ru_APTS(Me) and the diaminated support (Ru_AEAPTS), had higher TON, while the one with exclusively primary amines (Ru_APTS) had a lower TON. The diaminated system and the secondary amines also had a significantly higher conversion of CO_2_ into CH_3_OH or CO than the monaminated one. For Ru_AEAPTS, which showed the highest TON, we studied two different loadings of catalysts. Ru_AEAPTS_2, with the lower loading of supported molecular catalyst, gave a lower TON, but a higher selectivity to CO over CH_3_OH.

Besides CH_3_OH, CO and ammonia were observed in the gas‐phase IR spectra (Figure S5) used for the quantification, highlighted in Table 1. Diamines that are grafted on solid supports are less stable than their monoamine counterparts.^[34]^ This tendency could explain why more ammonia was generated from Ru_AEAPTS under the reaction conditions; however, the monoaminated support with secondary amines, Ru_APTS(Me), also yielded a high concentration of ammonia, which could limit its reusability (without any regeneration steps). Thermogravimetric analysis (Table S1) indicated that the amount of aminosilane fragments present during catalysis was approximately two orders of magnitude higher than the detected ammonia.

**Figure 2:**
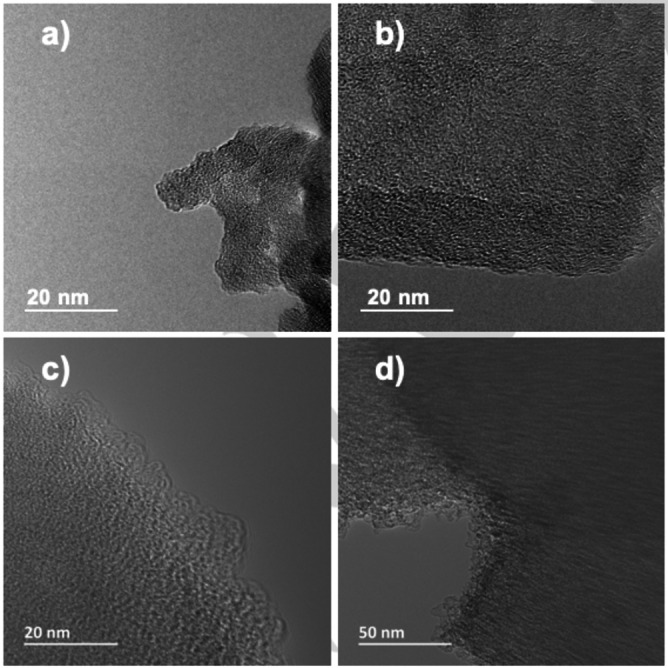
TEM images of Ru_APTS(Me) before (a,b) and after (c,d) reaction.

In order to try and understand the nature of the immobilized molecular catalyst, transmission electron microscopy images and solid‐state NMR spectra were recorded for the best‐performing composite (Ru_APTS(Me)) before and after hydrogenation of CO_2_. This was done to assess if there were any notable morphological or chemical changes in the composites after the reactions. As such, analysis of the TEM images for fresh (Figure 2a,b) and post‐catalysis (Figure 2c,d) Ru_APTS(Me) showed that the ruthenium species were evenly distributed on the silica support, and no agglomeration of ruthenium could be seen, which can be also observed in the EDS mapping (Figure S7). Interestingly, the reaction conditions do not change the apparent nature of the composite as the used samples present the same morphology under the TEM microscope.

**Figure 3:**
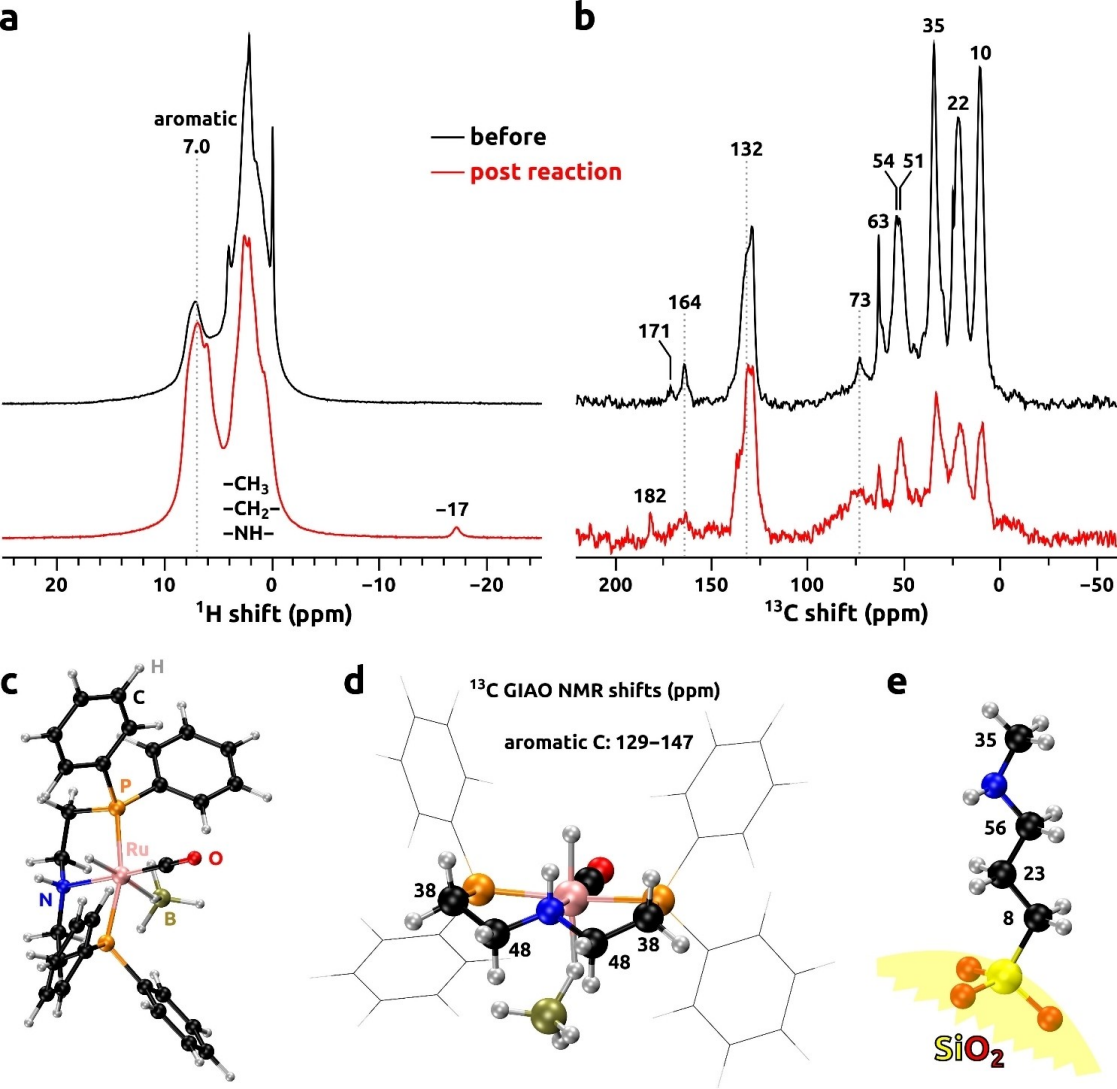
Solid‐state ^1^H MAS (a) and 1H‐^13^C CPMAS (b) NMR spectra of Ru_APTS(Me) catalyst before (black traces) and after reaction (red traces). Molecular geometry of the Ru_MACHOTM (c) shown together with the calculated ^13^C NMR shifts (d). Model of the APTS(Me) shown with calculated ^13^C NMR shifts (e).

Solid‐state ^1^H MAS as well as ^1^H‐^13^C CPMAS NMR spectra of the Ru_APTS(Me) catalyst were collected to assess the chemical nature and the integrity of the involved species. ^1^H MAS NMR spectra of the unused (Figure 3a, black trace) as well as the post‐reaction catalyst (Figure 3a, red trace) displayed two distinct groups of overlapping resonances. Signals in the chemical shift range from 0 to 4 ppm originated from protons in alkyl, methyl, and amine groups of Ru_MACHO^TM^ and APTS(Me).^[35]^ Signals between 6 and 10 ppm originated from the aromatic ligands of the Ru‐MACHO^TM^ catalyst (see Figure 3c for molecular structure). Interestingly, in the same sample, an additional ^1^H signal at around −17 ppm is observed. This signal could be attributed to the activation of the Ru center after the hydrogenation leading to the formation of a hydride.^[36]^

In the ^13^C NMR spectra shown in Figure 3b, several distinct resonances are observed. To provide assignments of these signals, ^13^C NMR shifts were calculated for the models of Ru_MACHO^TM^ and APTS(Me) at the DFT level using the previously validated protocol of Jaworski and Hedin,^[37]^ and results are shown in Figure 3d,e. ^13^C NMR signals at 10, 22, 35, and 54 ppm can be unambiguously assigned to the alkyl and methyl groups of the APTS(Me) (Figure 3e). Signals of alkyl carbon atoms from the Ru_MACHO^TM^ are observed at 51 ppm, and overlap with the signal at 38 ppm. Aromatic ligands of the Ru complex are responsible for a distinct group of resonances centered at 132 ppm. Based on our previous studies of amine‐modified silicas,^[35]^ signals between 150 and 200 ppm are most probably due to the interactions of the aminated support with CO_2_ and H_2_O. Signals at 63 and 73 ppm do not originate from the catalyst, and most probably are due to the presence of alcohol and methoxy groups in the sample from unreacted chains from the silanols used for the grafting, with the broadening in the post‐reaction samples attributed to the presence of different configurations of methanol that formed during the reaction and remained bound. In analogy to the ^1^H NMR data of Figure 3a, ^13^C NMR spectra reveal a relative increase of the aromatic signals compared to the alkyl/methyl spectral region.

## Conclusion

In conclusion, we highlight a heterogenized catalytic system consisting of aminated silica supports and the molecular catalyst, Ru‐MACHO^TM^, that is active in the hydrogenation of CO_2_ to CH_3_OH at a relatively low temperature of 155 °C. The most remarkable finding was the high TON values for the aminated catalytic systems containing secondary amines. The temperature used for the CO_2_ conversion in this study was significantly lower than for regular heterogeneous solid catalysts.^[33]^ Our catalytic conversion was almost three times higher than that observed by Pazdera et al.^[29]^ for the catalytic transformation of CO_2_ on Pd nanoparticles on monoaminated silica, while diaminated derivatives had not been studied. Notably, the product analysis in the present study was carried out using a gas‐phase IR analysis that allows the complete identification and quantification of the different species. We foresee that further experimentation and optimization of the reaction conditions for the systems investigated here could enable lower reaction temperatures and pressures, as well as allowing to study the reusability and long‐term stability of the system. When compared to other applied heterogeneous catalytic systems,^[33]^ our highest achieved conversion of 33 % is lower than the 66 % reached by using Cu‐ZnO/Al_2_O_3_.^[38]^ Our system, however, was able to convert CO_2_ to methanol at lower temperatures, that is, 155 °C instead of 260 °C. In addition, more in‐depth studies of the reaction mechanism with in situ knowledge of what is occurring during the reactions could help to understand the detailed chemistry. Such understanding could enable the design of the catalytic system to be more practical and applicable for industrial purposes.

## Experimental Section

### Materials and methods

Chromatographic particles of porous silica, Davisil LC60 (Grace Davison), were used as a support. They had particle sizes of 40–63 μm, and a surface area of 550 m^2^/g. The support was dried at 110 °C for 16 h. Toluene was analytical grade ([CAS: 108‐88‐3], Sigma‐Aldrich 99.8 %). The porous silica was modified with (3‐aminopropyl)triethoxysilane (APTS) (Sigma‐Aldrich >98 %), 3‐(methylamino)propyl)trimethoxysilane (APTS(Me)) (Sigma‐Aldrich >95 %) or 3‐(2‐aminoethylamino)propyltriethoxysilane (AEAPTS), (Sigma‐Aldrich >96 %). Anhydrous 1,4‐dioxane was used in the impregnation of the modified support with the Ru‐MACHO^TM^ molecular catalyst. H_2_ (≥99.999 %) was from Linde Gas and CO_2_ (>99.9 %) was from Strandmöllen AB.

The amine‐rich support was prepared using the methodology reported previously.[^35^, ^39^] In summary, for each synthesis, 3 g of dried porous silica and 180 cm^3^ of toluene were added to a three‐necked flask equipped with a Dean‐Stark reflux condenser. This was heated to 50 °C under stirring for 30 min; 0.3 mL of H_2_O was added and the mixture was refluxed for 1 h. After this period had elapsed, the required amount of silane monomer (APTS, APTS(Me) or AEAPTS; based on previous studies,^[40]^ five mol of silane per mol of free OH in the substrate) was added and the mixture was left to reflux for 24 h. The solid was filtered off and extracted in a Soxhlet extractor for 16 h with fresh toluene as solvent to ensure the removal of unreacted silanes. Finally, the solid was washed with toluene (2×50 cm^3^) and ethanol (3×50 cm^3^) and dried overnight at 110 °C. Following this grafting procedure, the amine‐modified silica was introduced into a glovebox, where it was added to a solution of Ru‐MACHO^TM^ catalyst in the minimum amount of 1,4‐dioxane to dissolve the Ru complex. The amount of solution was chosen to give m(Ru‐MACHO^TM^)/m(aminated silica+Ru‐MACHO^TM^)=0.05 (or 0.025 in the case of Ru_AEAPTS_2). The solution and modified support were stirred together inside the glovebox until the solvent had completely evaporated. The resulting pale yellow solid was stored in a vial in the glovebox until use.

### Thermogravimetric analysis

Thermogravimetric analysis (TGA) was used to record the mass loss on heating Silica_APTS, Silica_APTS(Me) and Silica_AEAPTS using a TA Instruments Discovery (TA Instruments, Stockholm, Sweden) thermobalance in dry air, for which samples were heated from 50 to 950 °C at a rate of 10 °C min^−1^ in a platinum cup.

### CO_2_‐adsorption measurements

Adsorption isotherms of CO_2_ on Ru_APTS, Ru_APTS(Me) and Ru_AEAPTS were measured at 0 °C using a Micrometrics ASAP2020 volumetric adsorption analyzer. Sample tubes were loaded in a glovebox under an inert N_2_ atmosphere, then pretreated under high dynamic vacuum conditions at 110 °C for 10 h. During measurement, thermal control was achieved by immersing the samples in a Dewar flask filled with ∼3 dm^3^ of H_2_O and ice, which equilibrated the temperature to 0 °C.

### CO_2_ hydrogenation

The reactions were carried out in a homemade fixed bed batch reactor (Figure S3). In a glovebox, 100 mg of catalyst was loaded in the reactor. The reactor was then flushed and pressurized to 2 bar CO_2_ and allowed to equilibrate for 2 h. Following this, the gas was switched to H_2_ and the pressure was increased to 75 bar and the temperature raised to 155 °C and held at that temperature for 40 h, then cooled to RT. In addition, the reaction was tested using a sample that consisted of APTS(Me) modified silica (without the presence of the Ru species) as well as the Ru species immobilized on unmodified silica.

### IR analysis of the gas phase

IR gas analysis was used to quantify the gas products. After the reaction, the cooled reactor was reheated to 100 °C and connected to the infrared gas cell. The cell was flushed with dry N_2_ and evacuated under a dynamic vacuum and the gas products from the reactor were subsequently collected into the cell for analysis.

The spectra were measured in the region of 600–4000 cm^−1^ with a spectral resolution of 0.5 cm^−1^. For quantification, a collection of quantitative gas‐phase IR spectra was used (QAsoft, Infrared Analysis Inc.). Reference spectra of known concentrations of certain compounds were spectrally subtracted from the measured spectra and the concentrations were calculated using the subtraction factor.^[41]^ Bands (or part of the bands) with intensities below 0.1 absorbance units were chosen for analysis in order to work in the linear region of the Lambert‐Beer law. Figure S4 contains reference spectra of the various compounds.

### Transmission electron microscopy (TEM)

TEM images were taken on a JEOL JEM‐2100F transmission electron microscope, equipped with a Schottky‐type field emission gun operating at an accelerating voltage of 200 kV.

### Solid state nuclear magnetic resonance (ss NMR)

^1^H magic‐angle spinning (MAS) NMR experiments were performed at a magnetic field of 14.1 T (^1^H Larmor frequency of 600.12 MHz) on a Bruker Avance‐III spectrometer with a 1.3 mm probehead and 60.00 kHz MAS rate. Acquisitions involved a rotor‐synchronized, double‐adiabatic spin‐echo sequence with a 90° excitation pulse of 1.25 μs followed by a pair of 50.0 μs tanh/tan short high‐power adiabatic pulses (SHAPs) with a 5 MHz frequency sweep. All pulses operated at a nutation frequency of 200 kHz. 128 signal transients with a 5 s relaxation delay were collected. ^1^H−^13^C cross‐polarization (CP) MAS experiments were performed at a magnetic field of 9.4 T (Larmor frequencies of 400.13, and 100.61 MHz for ^1^H and ^13^C, respectively) on a Bruker Avance‐III spectrometer with a 4 mm probehead and 14.00 kHz MAS rate. Experiments involved Hartmann‐Hahn‐matched ^1^H and ^13^C radiofrequency fields applied for 1.5 ms, SPINAL‐64 proton decoupling and 5 s relaxation delay. Chemical shifts were referenced with respect to neat tetramethylsilane (TMS). Models of the Ru_MACHO^TM^ and APTS(Me) were energy optimized at the revPBE‐D4/def2‐TZVP(Ru)/pcseg‐1 level of theory, and the ^13^C NMR shifts subsequently calculated at the PBE0/def2‐TZVP(Ru)/pcSseg‐1 level with the GIAO approach; in analogy to the protocol in Ref. [37]. Calculations were done with the ORCA code.^[42]^

## Supporting Information Summary

The Supporting Information contains TG curves, CO_2_ adsorption isotherms, details on the reactor and IR spectra from the reactions. Additional TEM images and EDS analysis is also included as well as data for the electronic structure calculations.

## Conflict of interest

The authors declare no conflict of interest.

1

## Supporting information

As a service to our authors and readers, this journal provides supporting information supplied by the authors. Such materials are peer reviewed and may be re‐organized for online delivery, but are not copy‐edited or typeset. Technical support issues arising from supporting information (other than missing files) should be addressed to the authors.

Supporting Information

## Data Availability

The data that support the findings of this study are available from the corresponding author upon reasonable request.
