# Supplementary material for: Precapture of CO2 and Hydrogenation into Methanol on Heterogenized Ruthenium and Amine‐Rich Catalytic Systems
Source: ChemistryOpen. 2023 May 31;12(6):e202300060. doi: 10.1002/open.202300060 (PMC10233216; doi:10.1002/open.202300060)
Supplement: Supplementary file 1 — Supporting Information [file OPEN-12-e202300060-s001.pdf]

ChemistryOpen

Supporting Information

Precapture of CO_2 and Hydrogenation into Methanol on Heterogenized Ruthenium and Amine-Rich Catalytic Systems

Anthony E. Szego, Tamara L. Church, Zoltán Bacsik, Aleksander Jaworski, Latif Ullah, and Niklas Hedin*

Contents

Materials 1
Catalyst preparation 1
Thermogravimetric analysis 2
CO_2-adsorption measurements 3
CO_2 hydrogenation 4
IR analysis of the gas phase products 5
Transmission electron microscopy 9
EDS Analysis 9
Electronic structure calculations 12

Materials

Chromatographic particles of porous silica, Davisil LC60 (Grace Davison), were used as a support. They had particle sizes of 40–63 µm, and a surface area of 550 m^2/g. The support was dried at 110 °C for 16 h. Toluene was analytical grade ([CAS: 108-88-3], Sigma-Aldrich 99.8%). The porous silica was modified with (3-aminopropyl)triethoxysilane (APTS) (Sigma-Aldrich >98%), 3-(Methylamino)propyl)trimethoxysilane (APTS(Me)) (Sigma-Aldrich >95%) or 3-(2-aminoethylamino)propyltriethoxysilane (AEAPTS), (Sigma-Aldrich >96%). Anhydrous 1,4-dioxane was used in the impregnation of the modified support with the Ru-MACHO^{TM} molecular catalyst. H_2 (≥99.999%) was from Linde Gas and CO_2 (>99.9%) was from Strandmöllen AB.

Catalyst preparation

The amine-rich support was prepared using the methodology reported previously.[35,39] In summary, for each synthesis, 3 g of dried porous silica and 180 cm^3 of toluene were added to a three-necked flask equipped with a Dean-Stark reflux condenser. This was heated to 50 °C under stirring for 30 min; 0.3 ml of H_2O was added and the mixture was refluxed for 1 h. After this period had elapsed, the required amount of silane monomer (APTS, APTS(Me) or AEAPTS; based on previous studies,[40] five moles of silane per mole of free OH in the substrate) was added and the mixture was left to reflux for 24 h. The solid was filtered off and extracted in a Soxhlet extractor for 16 h with fresh toluene as solvent to ensure the removal of unreacted silanes. Finally, the solid was washed with toluene (50 cm^3 x 2) and ethanol (50 cm^3 x 3) and dried overnight at 110 °C. Following this grafting procedure, the amine-modified silica was introduced into a glovebox, where it was added to a solution of Ru-MACHO^{TM} catalyst in the minimum amount of 1,4-dioxane to dissolve the Ru complex . The amount of solution was chosen to give m(Ru-MACHO^{TM})/m(aminated silica + Ru-MACHO^{TM}) = 0,05 (or 0,025 in the case of Ru_AEAPTS_2). The solution and modified support were stirred together inside the

glovebox until the solvent had completely evaporated. The resulting pale yellow solid was stored in a vial in the glovebox until used.

Thermogravimetric analysis

Thermogravimetric analysis (TGA) was used to record the mass loss on heating Silica_APTS, Silica_APTS(Me) and Silica_AEAPTS using a TA Instruments Discovery (TA Instruments, Stockholm, Sweden) thermobalance in dry air, for which samples were heated from 50 to 950 °C at a rate of 10 °C min^{-1} in a platinum cup.

This was done to estimate the amount of aminated fragments grafted on each modified silica sample used as a support. The temperature decomposition profiles can be seen in figure S1. Analysis of the mass lost between 200 and 850 °C was used to obtain the data compiled in Table S1.

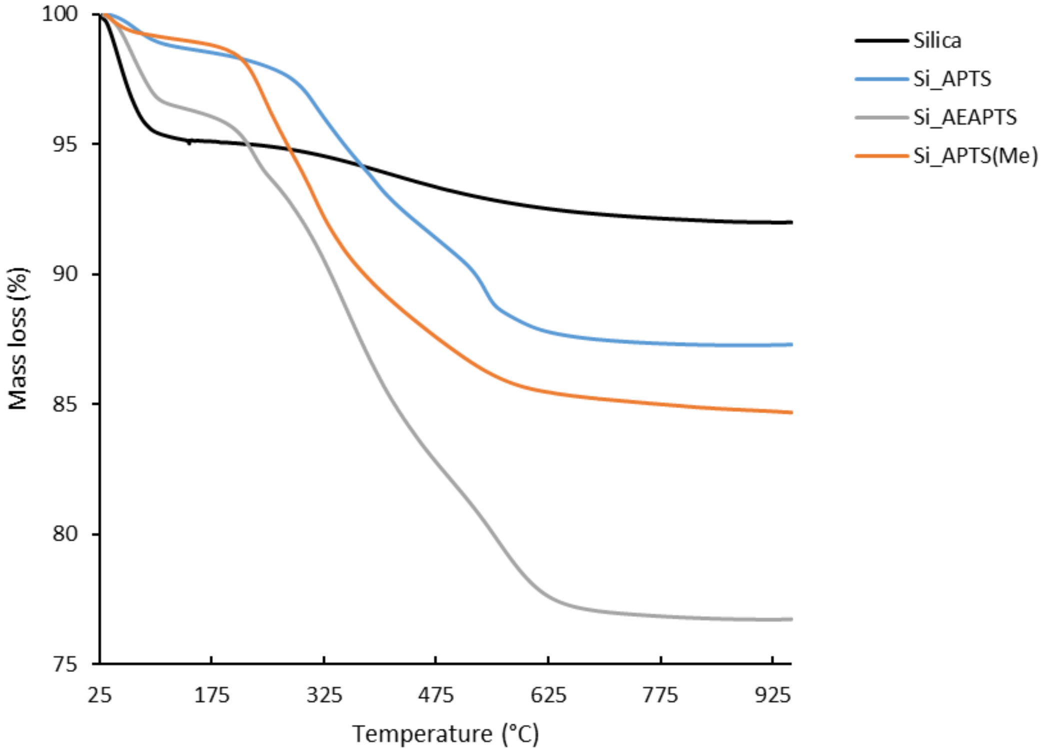

Figure S1. Temperature decomposition profiles of the amine-modified silica samples in dry air.

Table S1. Amount of monomer fragments present in 100 mg of each of the modified silica samples calculated from the TGA curves.

Sample	Weight loss due to organics (%)	Amount of monomer fragment (mmol 100 mg of sample)
Silica_APTS	11	0.19
Silica_APTS(Me)	13	0.18
Silica_AEAPTS	21	0.21

CO_2-adsorption measurements

Adsorption isotherms of CO_2 on Ru_APTS, Ru_APTS(Me) and Ru_AEAPTS were measured at 0 °C using a Micrometrics ASAP2020 volumetric adsorption analyzer. Sample tubes were loaded in a glovebox under an inert N_2 atmosphere, then pretreated under high dynamic vacuum conditions at 110 °C for 10 h. During measurement, thermal control was achieved by immersing the samples in a Dewar flask filled with ~3 dm^3 of H_2O and ice, which equilibrated the temperature to 0 °C.

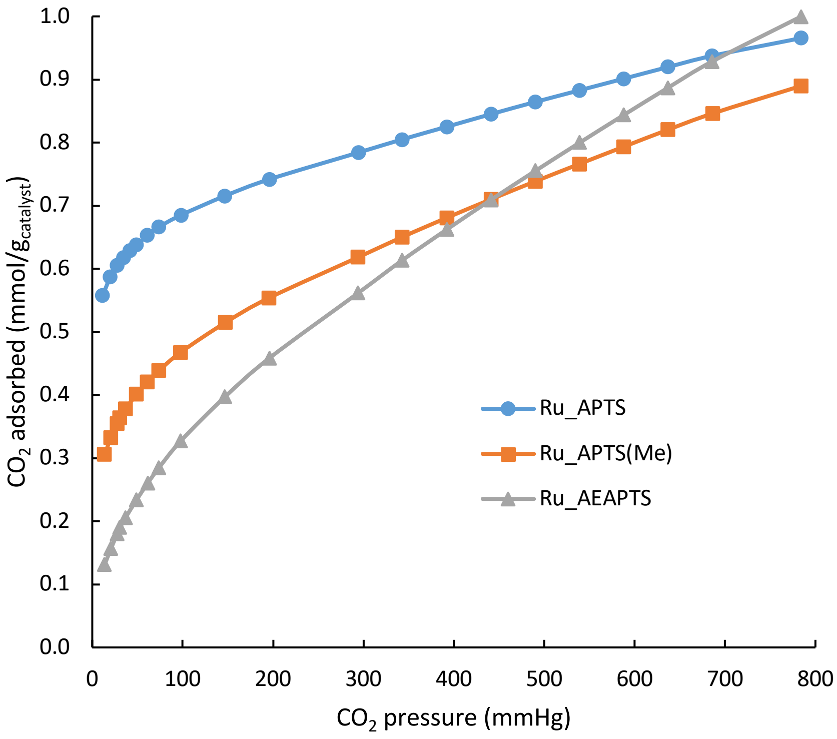

Figure S2. CO_2 adsorption isotherms at 0 °C for the supported, amine-modified catalysts.

CO_2 hydrogenation

The reactions were carried out in a homemade fixed bed batch reactor (Figure S3). In a glovebox, 100 mg of catalyst was loaded in the reactor. The reactor was then flushed and pressurized to 2 bar CO_2 and allowed to equilibrate for 2 h. Following this, the gas was switched to H_2 and the pressure was increased to 75 bar and the temperature raised to 155 °C and held at that temperature for 40 h, then cooled to RT.

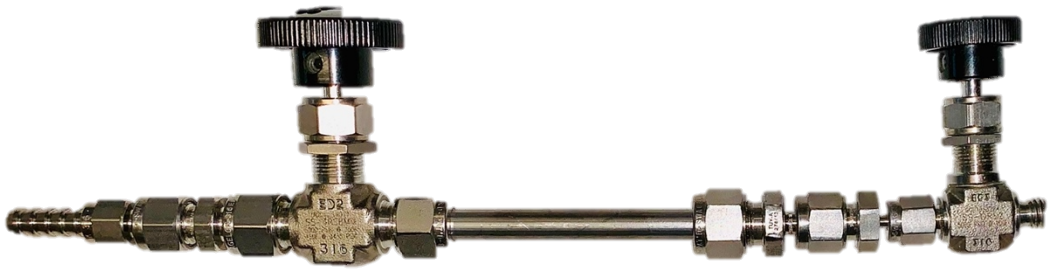

Figure S3. Reactor used for the hydrogenation reactions.

Turnover Numbers (TON) were calculated using the following equation:

$$TON = \frac{moles\ of\ MeOH\ formed}{moles\ of\ supported\ molecular\ catalyst\ in\ the\ reaction}$$

Conversion was calculated based on the initial 2 bar of CO_2 added to the reactor:

$$Conversion\ (\%) = \frac{moles\ of\ MeOH\ formed}{intial\ moles\ of\ CO_2} * 100$$

Selectivity to CO and methanol was calculated taking into account the amount of products formed:

$$Selectivity\ to\ X\ (\%) = \frac{moles\ of\ product\ X\ formed}{total\ moles\ of\ products\ formed} * 100$$

IR analysis of the gas phase products

IR gas analysis was used to quantify the gas products. After the reaction, the cooled reactor was reheated to 100 ^{o}C and connected to the infrared gas cell. The cell was flushed with dry N_2 and evacuated under a dynamic vacuum and the gas products from the reactor were subsequently collected into the cell for analysis.

The spectra were measured in the region of 600–4000 cm^{-1} with a spectral resolution of 0.5 cm^{-1}. For quantification, a collection of quantitative gas-phase IR spectra was used (QAsoft, Infrared Analysis Inc.). Reference spectra of known concentrations of certain compounds were spectrally subtracted from the measured spectra and the concentrations were calculated using the subtraction factor.[41] Bands (or part of the bands) with intensities below 0.1 absorbance units were chosen for analysis in order to work in the linear region of Lambert-Beer law. Figure S4 contains reference spectra of the various compounds.

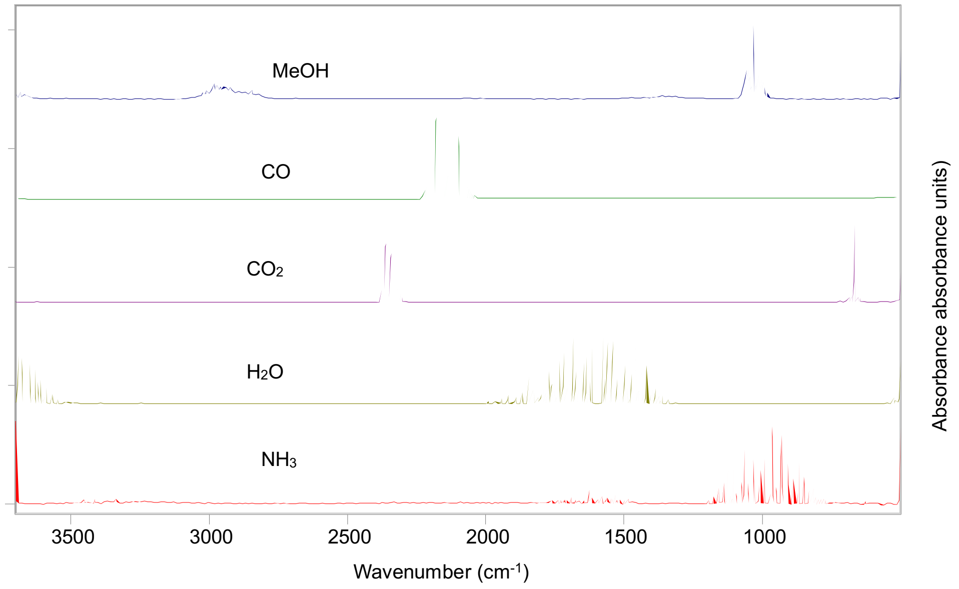

Figure S4. IR reference spectra for involved gases.

Figure S5 shows example spectra for the gas phase after reaction over each catalyst.

a)

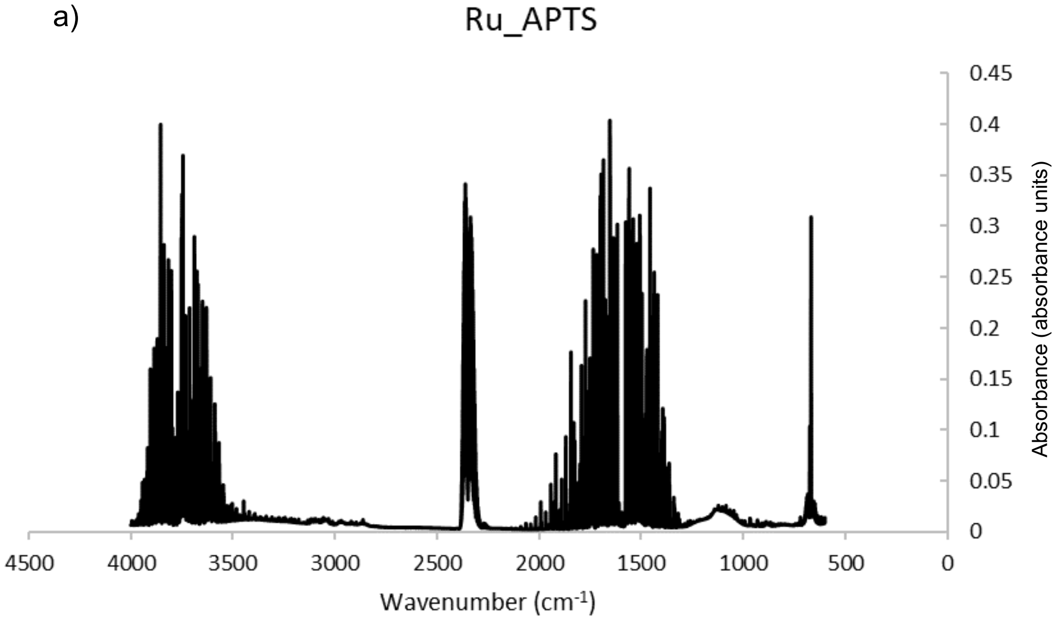

b) Ru_APTS(Me)

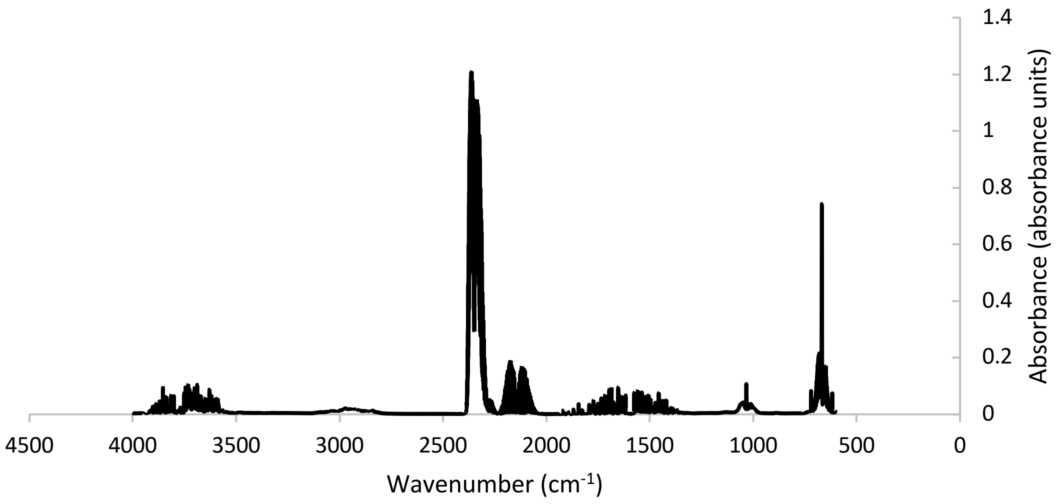

c) Ru_AEAPTS

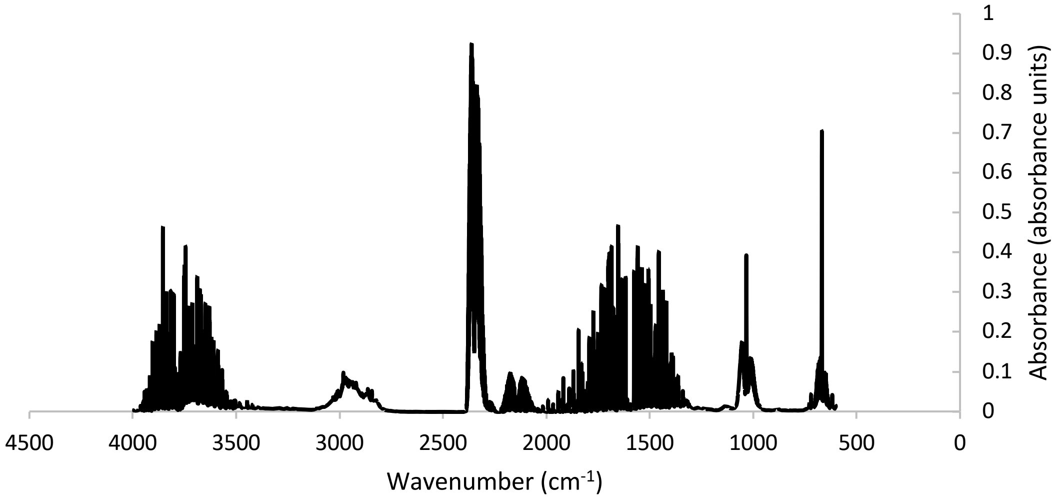

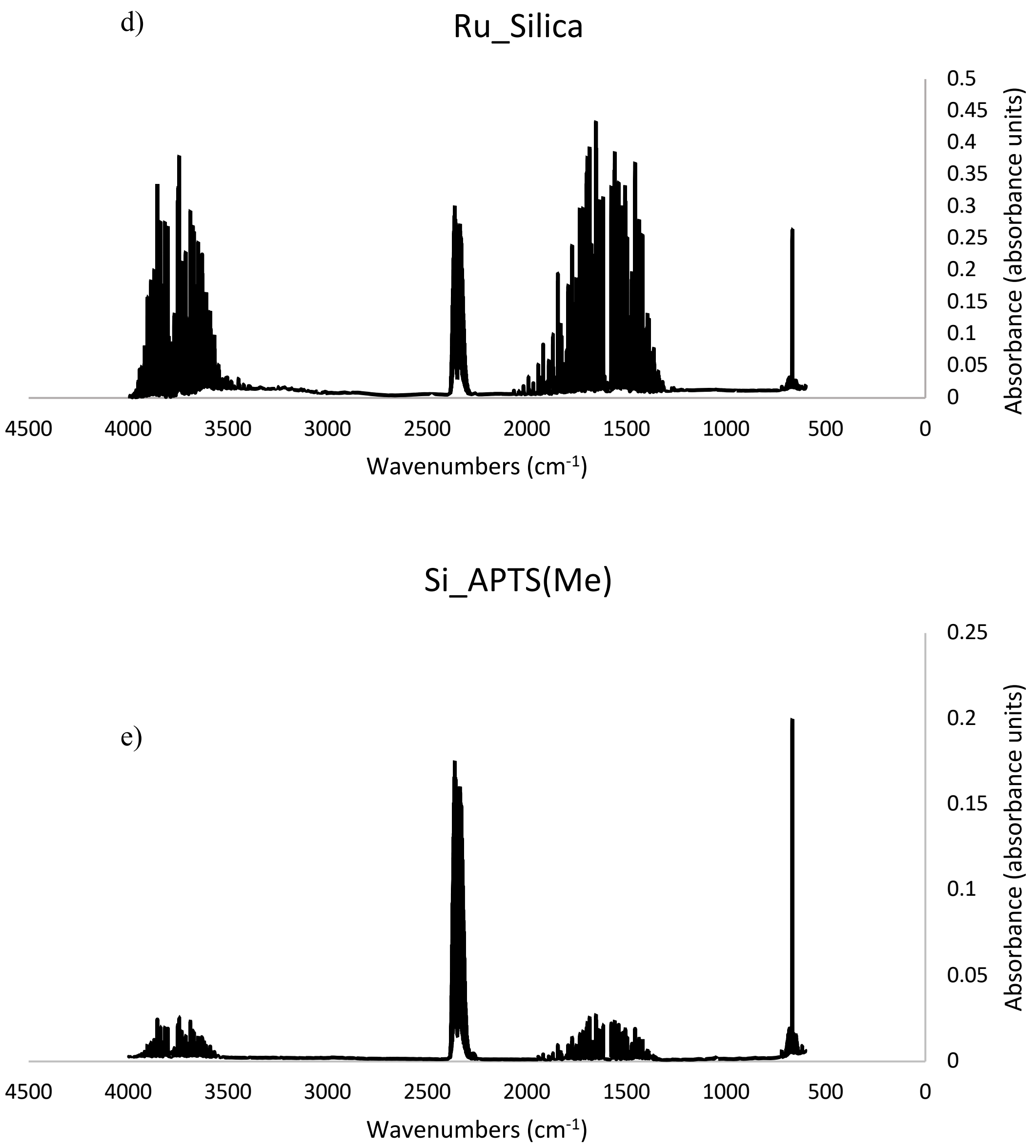

Figure S5. Example spectra for the gas phase after reaction over each catalyst. The reactor was subjected to 2 bar CO_2 for 2 h at room temperature. The pressure was then switched to 75 bar H_2 and the reactor was heated to 155 °C and held at that temperature for 40 h, then cooled to room temperature to stop the reaction and the gases were extracted into the cell with vacuum and N_2 flushing at 100 °C.

Transmission electron microscopy

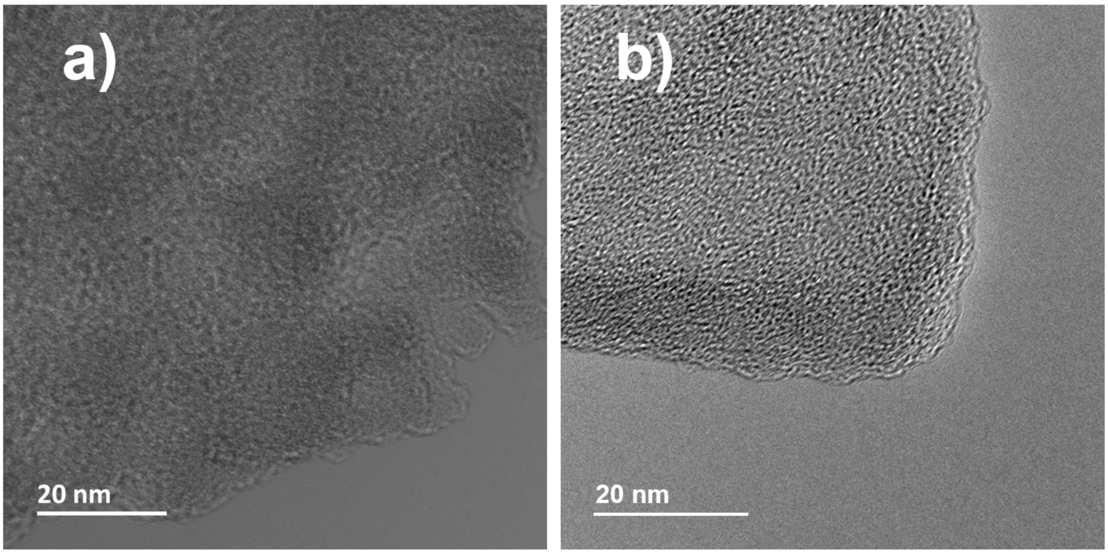

Figure S6. TEM images of a) Ru_APTS, and b) Ru_AEAPTS (before reaction).

EDS Analysis

The EDS analyses were done on Hitachi TM3000 scanning electron microscope, equipped with a thermionic-type filament (accelerating voltage 5 - 15 kV) and Bruker Quantax 70 EDS detector having resolution of 135 eV. The powder sample was mounted on a sticky conducting carbon disc and measured at a magnification of 800x.

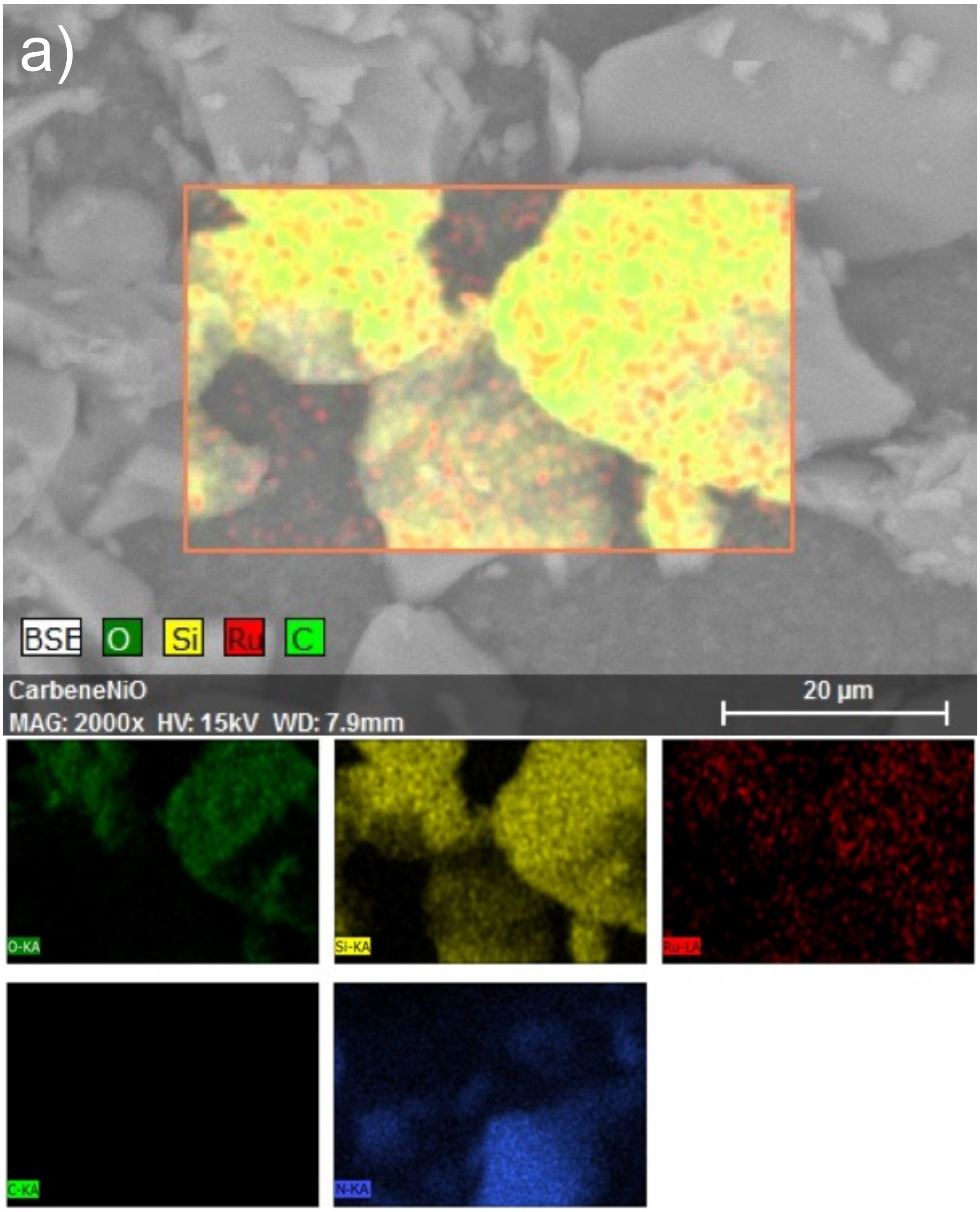
a)
BSE
O
Si
Ru
C
CarbeneNiO
MAG: 2000x HV: 15kV WD: 7.9mm
20 µm
O-KA
Si-KA
Ru-LA
C-KA
N-KA

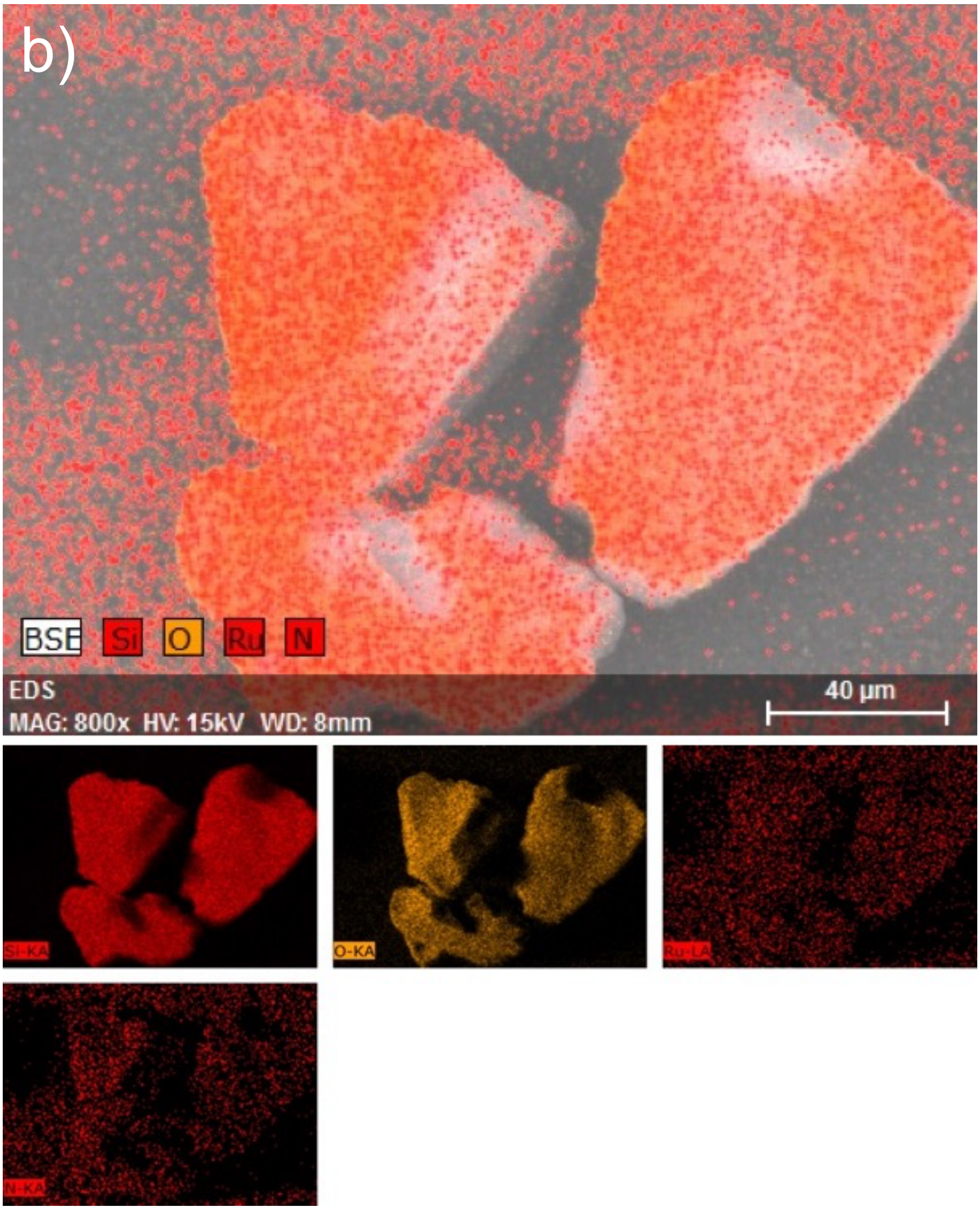

Figure S7. EDS mapping of Ru_APTS(Me) a)before reaction and b) after reaction.

Electronic structure calculations

Table S2. Energy optimized geometries (revPBE-D4/def2-TZVP level of theory) of the models used in the calculations

Ru-MACHO^{TM} Total Energy: -2057.40841487 Hartree										
Cartesian coordinates (Å)				Internal coordinates (Å)						
Ru	0.271690	-0.713986	-0.417474	Ru	0	0	0	0.000000000000	0.00000000	0.00000000
P	-1.781144	-1.760926	-0.450997	P	1	0	0	2.304633199372	0.00000000	0.00000000
P	2.013923	0.739878	-0.046855	P	1	2	0	2.299229226977	164.69891172	0.00000000
N	-0.962673	1.077288	-0.009875	N	1	2	3	2.213244650277	82.73622994	336.91014535
C	1.290678	-2.225206	-0.625364	C	1	2	4	1.834486577708	96.82865566	175.84626932
O	1.940832	-3.200939	-0.724619	O	5	1	2	1.176692548472	178.30261392	279.01711509
C	1.133897	2.364552	0.295207	C	3	1	2	1.879101011745	102.77857172	335.98718420
C	-0.254209	2.348073	-0.342683	C	4	1	2	1.492506871975	112.53865416	201.09152627
C	-2.304625	0.981736	-0.653867	C	4	1	2	1.491540188256	111.74143080	329.01855678
C	-2.986856	-0.325413	-0.258372	C	9	4	1	1.526595471683	110.18631306	53.06570805
H	1.721309	3.217601	-0.084730	H	7	3	1	1.103221411578	110.88477700	213.70052242
H	1.070217	2.474045	1.393706	H	7	3	1	1.105777220975	107.06273761	97.03636820
H	-0.170680	2.380439	-1.440648	H	8	4	1	1.101612858177	106.45465262	69.47063190
H	-0.852410	3.216733	0.002510	H	8	4	1	1.109763183578	109.95194150	187.56651165
H	-2.126985	1.028695	-1.741146	H	9	4	1	1.102694963422	106.14443991	292.39741772
H	-2.931146	1.850209	-0.361856	H	9	4	1	1.109975470392	110.12839282	175.16572784
H	-3.895080	-0.490848	-0.862805	H	10	9	4	1.103439203998	110.75045049	189.98812077
H	-3.288820	-0.308797	0.805914	H	10	9	4	1.106419091892	110.99663714	70.36958216
C	-2.370424	-2.682698	-1.927936	C	2	1	3	1.838004468708	121.67432530	144.61832469
C	-2.333959	-2.029036	-3.176596	C	19	2	1	1.409878168893	118.09040828	308.56190746
C	-2.750821	-2.704238	-4.330832	C	20	19	2	1.400689888043	119.90935065	178.82867883
C	-3.183306	-4.037944	-4.255904	C	21	20	19	1.404075069225	120.37026862	358.87109039
C	-3.190822	-4.698932	-3.019343	C	22	21	20	1.402158127506	119.76837457	359.66009050
C	-2.786447	-4.026367	-1.856535	C	23	22	21	1.402848869192	120.22826460	0.91788638
H	-1.944905	-1.007662	-3.255816	H	20	19	2	1.095830194686	120.35371287	1.18482849
H	-2.717342	-2.187869	-5.297317	H	21	20	19	1.096289452779	119.36269532	180.08866101
H	-3.500141	-4.566103	-5.163131	H	22	21	20	1.096538092714	120.15687217	180.56442502
H	-3.509581	-5.746294	-2.955298	H	23	22	21	1.096665717864	120.21691341	181.13054628
H	-2.789531	-4.552132	-0.896542	H	24	23	22	1.094542587069	119.72846353	180.38374265
C	-2.269906	-2.866562	0.934370	C	2	1	3	1.838630218553	119.93374318	275.29204306
C	-1.280732	-3.453567	1.741993	C	30	2	1	1.405452975917	119.80418208	337.26711554
C	-1.645704	-4.309572	2.792601	C	31	30	2	1.403468916824	120.12410643	180.66102328
C	-2.998686	-4.575215	3.048662	C	32	31	30	1.402388580007	120.20553495	359.49389358
C	-3.991403	-3.987327	2.247679	C	33	32	31	1.404518597689	119.89993664	0.27205516
C	-3.629079	-3.140902	1.191692	C	34	33	32	1.401007363483	119.99573259	0.23547958
H	-4.408992	-2.702939	0.557855	H	35	34	33	1.096278321467	119.54863063	178.70281686
H	-5.050652	-4.192572	2.443958	H	34	33	32	1.096658036995	120.15127082	179.97204720
H	-3.282739	-5.239549	3.873531	H	33	32	31	1.096556154475	120.13396670	180.35997681
H	-0.867724	-4.765367	3.416425	H	32	31	30	1.096430416460	119.66577565	179.85141079
H	-0.228131	-3.221842	1.549456	H	31	30	2	1.094868064859	119.15621146	1.58479745

C	3.035525	0.497694	1.471983	C	3	1	2	1.846400018850	117.96380851	88.26198813
C	2.387334	0.426418	2.721350	C	41	3	1	1.409307511483	118.77640590	306.76145067
C	3.119298	0.202620	3.893048	C	42	41	3	1.399547730750	120.64554301	178.02830468
C	4.510639	0.020937	3.829314	C	43	42	41	1.404599733115	120.05028360	358.78145776
C	5.158775	0.064557	2.587698	C	44	43	42	1.401283254338	119.62638548	359.96872062
C	4.427745	0.305003	1.413342	C	45	44	43	1.404039942823	120.37236000	0.80014446
H	1.296204	0.518016	2.774164	H	42	41	3	1.096240799918	119.75390364	359.80995295
H	2.600872	0.155197	4.858275	H	43	42	41	1.096666550028	119.77095684	179.66894308
H	5.084023	-0.163603	4.745393	H	44	43	42	1.096368582921	120.12619132	180.56070423
H	4.944091	0.338253	0.448816	H	46	45	44	1.094544911207	119.77791133	179.92477453
H	6.242630	-0.089789	2.526977	H	45	44	43	1.096472629826	120.12536283	180.83283428
C	3.287485	1.084039	-1.317074	C	3	1	2	1.831355838277	122.27485428	216.36452296
C	3.476395	0.143877	-2.344013	C	52	3	1	1.405060577688	118.33921274	338.52776902
C	4.477224	0.345900	-3.303317	C	53	52	3	1.400977187794	120.00167197	182.54861086
C	5.286347	1.489993	-3.247390	C	54	53	52	1.402410876819	120.16525407	359.55149794
C	5.097042	2.434207	-2.225443	C	55	54	53	1.404191703854	119.98741324	0.20715090
C	4.103029	2.229805	-1.259145	C	56	55	54	1.401275268411	119.96128949	0.24872130
H	3.976388	2.960773	-0.451558	H	57	56	55	1.096608135333	119.50679149	178.64779058
H	2.818304	-0.727666	-2.404241	H	53	52	3	1.093754163442	119.50731012	4.35724111
H	4.612017	-0.386787	-4.107256	H	54	53	52	1.096046118441	119.58463500	180.61128844
H	6.063042	1.651497	-4.004378	H	55	54	53	1.096526185256	120.08574554	180.57651834
H	5.726630	3.330874	-2.179675	H	56	55	54	1.096579599418	120.18619766	180.11278778
H	0.115761	-0.447070	-2.193324	H	1	2	19	1.802554484521	88.61813022	30.58121939
H	-1.100935	1.048592	1.009680	H	4	1	2	1.029287350552	103.57838296	84.75041611
B	0.213855	0.490894	-3.116239	B	63	1	2	1.319533927365	141.98876733	130.27189522
H	-0.946420	0.855324	-3.298849	H	65	63	1	1.229794609116	104.14411179	262.37324143
H	0.956118	1.403807	-2.801771	H	65	63	1	1.217889299206	113.42437988	25.66297292
H	0.656004	-0.190255	-4.029977	H	65	63	1	1.222446682987	98.83621069	145.51359288
H	0.273338	-0.959918	1.176237	H	1	2	19	1.612575073159	86.90483015	208.56666424
APTSMe Total Energy: -730.00835840 Hartree										
Cartesian coordinates (Å)				Internal coordinates (Å)						
Si	-0.339407	-1.406059	0.279304	Si	0	0	0	0.000000000000	0.00000000	0.00000000
O	-1.859077	-2.091537	0.122907	O	1	0	0	1.674436050257	0.00000000	0.00000000
O	0.884268	-2.285779	-0.483051	O	1	2	0	1.688926323947	113.71365920	0.00000000
O	0.111412	-1.440562	1.894200	O	1	2	3	1.676996344920	108.99583738	245.42119474
C	-0.491000	0.321846	-0.413465	C	1	2	3	1.867770473514	105.68914481	123.14975659
H	-1.964465	-2.920973	0.627067	H	2	1	3	0.976343647319	113.42286945	69.72847086
H	0.802222	-2.335037	-1.453567	H	3	1	2	0.975223187815	114.49160975	64.65145651
H	1.051330	-1.669810	2.020291	H	4	1	2	0.975653834288	112.84657115	139.62574700
H	-0.888437	0.255570	-1.446827	H	5	1	2	1.109137826799	108.62481952	304.98231376
C	0.827998	1.111964	-0.385959	C	5	1	2	1.537790322256	113.52046291	181.27725685
H	-1.270776	0.847563	0.172522	H	5	1	2	1.108066060856	107.45065838	58.77223681
H	1.600804	0.566011	-0.965301	H	10	5	1	1.109473193543	109.58931669	58.45867867
H	1.213615	1.181357	0.648865	H	10	5	1	1.106515154840	110.35669846	302.03313711
C	0.692484	2.530902	-0.946462	C	10	5	1	1.531637562544	113.17624069	180.41243149
H	0.282215	2.481038	-1.988432	H	14	10	5	1.120940930938	109.35013852	303.90353208

N 1.959060 3.257674 -0.860208<br>H -0.058628 3.088981 -0.351334<br>H 2.671507 2.740817 -1.390629<br>C 1.878385 4.622616 -1.377424<br>H 1.494073 4.703060 -2.424185<br>H 2.876983 5.093339 -1.338686<br>H 1.204934 5.219739 -0.733734	N 14 10 5 1.462822894455 111.22835697 178.15792054<br>H 14 10 5 1.108961851618 109.25377823 59.34094740<br>H 16 14 10 1.027651725012 108.66025523 59.04577771<br>C 16 14 10 1.461878869264 113.28092769 180.31269076<br>H 19 16 14 1.117977969297 114.67171675 305.27692670<br>H 19 16 14 1.104662256688 109.60743324 183.32626087<br>H 19 16 14 1.106539226041 109.36732785 65.77177590
CH4 Total Energy: -40.49366303 Hartree	
Cartesian coordinates (Å)	Internal coordinates (Å)
C 0.533653 0.399841 -0.680220<br>H -0.082391 0.497416 -1.589799<br>H -0.029016 -0.157838 0.087106<br>H 0.783926 1.402870 -0.295906<br>H 1.462606 -0.143289 -0.922101	C 0 0 0 0.000000000000 0.00000000 0.00000000<br>H 1 0 0 1.102889446567 0.00000000 0.00000000<br>H 1 2 0 1.102900882321 109.48477889 0.00000000<br>H 1 2 3 1.102905479565 109.49181409 239.98943262<br>H 1 2 3 1.102928599307 109.46084004 119.99753953
